# Effect of a community-based intervention for cardiovascular risk factor control on stroke mortality in rural Gadchiroli, India: a cluster-randomised controlled trial

**DOI:** 10.12688/wellcomeopenres.26002.2

**Published:** 2026-07-08

**Authors:** Yogeshwar Kalkonde, Sunil Jadhao, Vidya Bhusari, Sarita Logade, Sindhu Nila, Shoummo Sen Gupta, Umesh Jadhav

**Affiliations:** 1 Stroke Trial Project Gadchiroli, Gadchiroli, Maharashtra, India; 2 Sangwari, People's Association for Equity and Health, Ambikapur, Chhattisgarh, India

**Keywords:** stroke, prevention, mortality, hypertension, diabetes, treatment, trial, rural, India, community health worker

## Abstract

**Background:**

Stroke is the second leading cause of death globally. We tested whether it was feasible to deliver a community-based intervention package for prevention of stroke and, whether such an intervention would reduce stroke mortality in a rural underserved community in central India.

**Methods:**

In this two-arm, parallel group, open label, cluster randomised controlled trial, 64 villages were randomly assigned with 1:1 allocation to the control and the intervention arm in a demographic surveillance site. The intervention was delivered for 3.5 years. It included community-based screening for hypertension, diabetes and stroke among individuals ≥ 50 years of age by trained community health workers followed by village-based treatment by an outreach physician, awareness generation and active follow up. In the control arm, households were provided information on the ill effects of tobacco and steps to quit it. Cause of death was ascertained on all deaths using verbal autopsies by physician coders who were blinded to the treatment allocation.

**Results:**

There were 7671 individuals in the control arm and 8255 in the intervention arm who were ≥ 50 years of age. In the intervention arm, out of 8255 eligible individuals, 8026 were screened and 2793 were diagnosed with hypertension, 317 with diabetes and 182 with stroke and those with these three conditions received the intervention. The stroke mortality rate per 100,000 person-years in the last 2.5 years of the intervention, which was the primary outcome of the trial, was comparable in the intervention (416.7) and the control arm (484.9) (adjusted hazard ration [HR] 0.84, 95 % confidence interval [0.62,1.12]; p = 0.23). Important limitations include limited access to brain imaging and lipid profile measurements.

**Conclusions:**

In this under resourced rural community in central India, a community-based intervention for stroke risk reduction did not reduce stroke mortality over the final 2.5 years of the trial. However, the trial intervention was acceptable and led to improved medication compliance, blood pressure control and awareness about stroke.

**Trial registration:**

The trial was registered at the Clinical Trials Registry of India: CTRI/2015/12/006424.

## Plain Language summary

Stroke is the second leading cause of death worldwide, and many people do not have their risk factors such as high blood pressure and diabetes under good control. We tested if a community-based program could be delivered in a rural setting in central India and whether it could reduce deaths from stroke. We conducted a trial in which one group of villages received a community-based preventive program for 3.5 years, while another group did not. We then compared stroke deaths between these two groups during the last 2.5 years of the program. In the intervention villages, trained community health workers screened adults aged 50 and above for high blood pressure, diabetes, and stroke symptoms. Those who screened positive were examined and treated by an outreach doctor. Health workers visited homes every month to check blood pressure, ensure people took their medicines, and give advice on reducing tobacco, alcohol, and salt. After 2.5 years, stroke deaths were lower in the intervention villages, but the difference was not statistically significant. The program did successfully reduce blood pressure and improve stroke awareness. It did not reduce blood sugar levels or the use of tobacco or alcohol. When we looked at the full 3.5-year trial period, stroke deaths were significantly lower in the intervention villages. Although the reduction in stroke deaths was not significant over the last 2.5 years, the improvement in awareness and blood pressure control suggests that community-based prevention of stroke with an active role for community health workers and making treatment available in villages could be included in India’s national program for prevention and control of non-communicable diseases (NP-NCD). The main limitations of the study were the lack of access to certain tests, such as brain scans, cholesterol testing, and measurements to assess long term blood sugar control.

## Background

Globally, stroke is the second leading cause of death and the third leading cause of death and disability combined together. ^1, 2^ It is no longer a disease of high-income countries as low- and middle-income countries (LMICs) now account for 75% of all stroke deaths that occur worldwide. ^3^ A large population in these countries lives in rural areas where access to curative and preventive care for stroke is poor leading to higher mortality and morbidity. Stroke has emerged as a major public health problem in rural India. In community-based studies it was a leading cause of death and accounted for 12-14% of all deaths. ^4, 5^ In a nationally representative study, stroke mortality was higher in rural than urban India. ^6^ Interventions are therefore urgently needed to reduce the mortality due to stroke in rural India.

There are several challenges to stroke care in rural India. Patients with acute stroke often do not receive modern medical care and have preference for traditional medicines. ^7, 8^ Prevention of stroke thus becomes imperative until such facilities and care seeking become established. The INTERSTROKE and the Global Burden of Disease study (GBD) have found that 90% of the risk of stroke is due to modifiable risk factors. ^9, 10^ Among these, hypertension is the leading risk factor and accounts for close to 50% of the risk of stroke. ^10^ But control of hypertension remains poor in rural India and only about 10% of patients have adequately controlled blood pressure. ^11, 12^ Diabetes, another risk factor for stroke, also remains poorly controlled and only 30% of patients with diabetes in rural India achieve adequate control. ^13^ There are also challenges to the secondary prevention of stroke which includes treatment with statins and anti-thrombotic medications as well as management of risk factors such as hypertension, diabetes and atrial fibrillation after stroke. In a study in rural Gadchiroli we found that < 10% of stroke patients were receiving aspirin or statins. ^14^ Collectively, these studies highlight a significant gap in prevention of stroke in rural India where 12% of the world’s population lives. ^15^

The emergence of stroke as a public health priority in Gadchiroli prompted us to seek a solution. ^4^ We hypothesized that a community-based health care delivery intervention package to address major local barriers to effective control of hypertension, diabetes and secondary prevention of stroke in a rural under resourced setting in India will reduce stroke mortality.

## Methods

### Study design and setting

This community-based, two-arm, parallel-group, superiority, open-label cluster randomised controlled trial was conducted in 64 villages (clusters) in Gadchiroli district, Maharashtra, India with 1:1 allocation of villages to control and intervention arms. As the intervention was community-based, a cluster randomized design was chosen. The study protocol was published previously. ^16^

Gadchiroli, located in central India is among the country’s most underdeveloped districts. ^17^ It has a population of about one million, 90% residing in rural areas. Agriculture and manual labour are the predominant occupations. ^18^

The government health care system is an important source of health care in the district and provides care through one district hospital, 12 rural or sub-district hospitals, 48 primary health centres and 34 sub-health centres. ^19^ When the trial intervention started, facilities for diagnosis and treatment of hypertension and diabetes were available at the district and block level hospitals in the public health system. Free medications were available in these facilities and were dispensed for a period of 7-15 days after each consultation. The National Programme for Prevention and Control of Cancer, Diabetes, Cardiovascular Diseases, and Stroke (NPCDCS)-now the National Programme for Prevention and Control of Non-Communicable Diseases (NP-NCD)-introduced village-level screening for hypertension, diabetes, and cancer through community health worker cadre called the Accredited Social Health Activists (ASHAs) and Auxiliary Nurse Midwives (ANMs) followed by referral to nearest public health facility for treatment. ^20^ Treatment for hypertension (amlodipine, telmisartan, chlorthalidone in the state of Maharashtra where this trial was conducted) and diabetes (metformin and glimepiride) were made available at the peripheral government run hospitals such as the primary health centres (serving a population of about 20000 to 30000) and health and wellness centres (serving a population of about 3000-5000). The screening activities by the ASHAs/ANMs and treatment activities at the health and wellness centres under this programme gradually became operational in the intervention as well as control villages under the trial in the second half of 2018. However, the process of implementation of screening as well as availability of treatments was variable across these villages during the trial period. In addition to government-run services, healthcare was also available through formal and informal (unqualified, unlicensed) private providers and a few non-governmental organizations. At the study onset, the district had only one computerized tomography (CT) scanner and by the end of the trial one more additional CT scanner became available in a private facility. The nearest facilities for giving tissue plasminogen activator (t-PA) for acute ischemic stroke and neurosurgical care for selected patients with intracerebral hemorrhage was available in a private set up in the neighbouring district which was 100 kilometres away from Gadchiroli town.

The trial was conducted in the field service area of a local non-profit organization, which maintained a demographic surveillance system (DSS) with a population register across 86 villages in 3 geographic administrative blocks of the district (Gadchiroli, Armori and Chamorshi). ^16^ The DSS systematically recorded all deaths, and the cause of death was determined through verbal autopsies. ^4^ The organization operated a rural hospital providing primary and secondary level outpatient and inpatient care at a nominal cost.

### Inclusion and exclusion criteria

Villages were included in the study if a) they were in the service area of the host organization, and b) had population >400 so that the average village size of the selected villages is close to the average village population in India which is about 1250. ^15^ Villages within five kilometers of Gadchiroli town were excluded as the purpose of the study was to assess the impact of the intervention in a larger rural community which has difficulty in accessing healthcare. Individuals from the selected intervention villages were eligible to be recruited if they were resident of the village for >6 months and were ≥50 years of age at the time of screening. A list of individuals ≥50 years of age at the time of commencement of the trial was drawn from the population register. Terminally ill individuals were excluded from receiving the trial intervention. Those diagnosed with hypertension, diabetes or stroke were eligible to receive the intervention and the operational definitions for these conditions is provided in the procedures section.

### Randomisation and blinding

**Figure 1.**
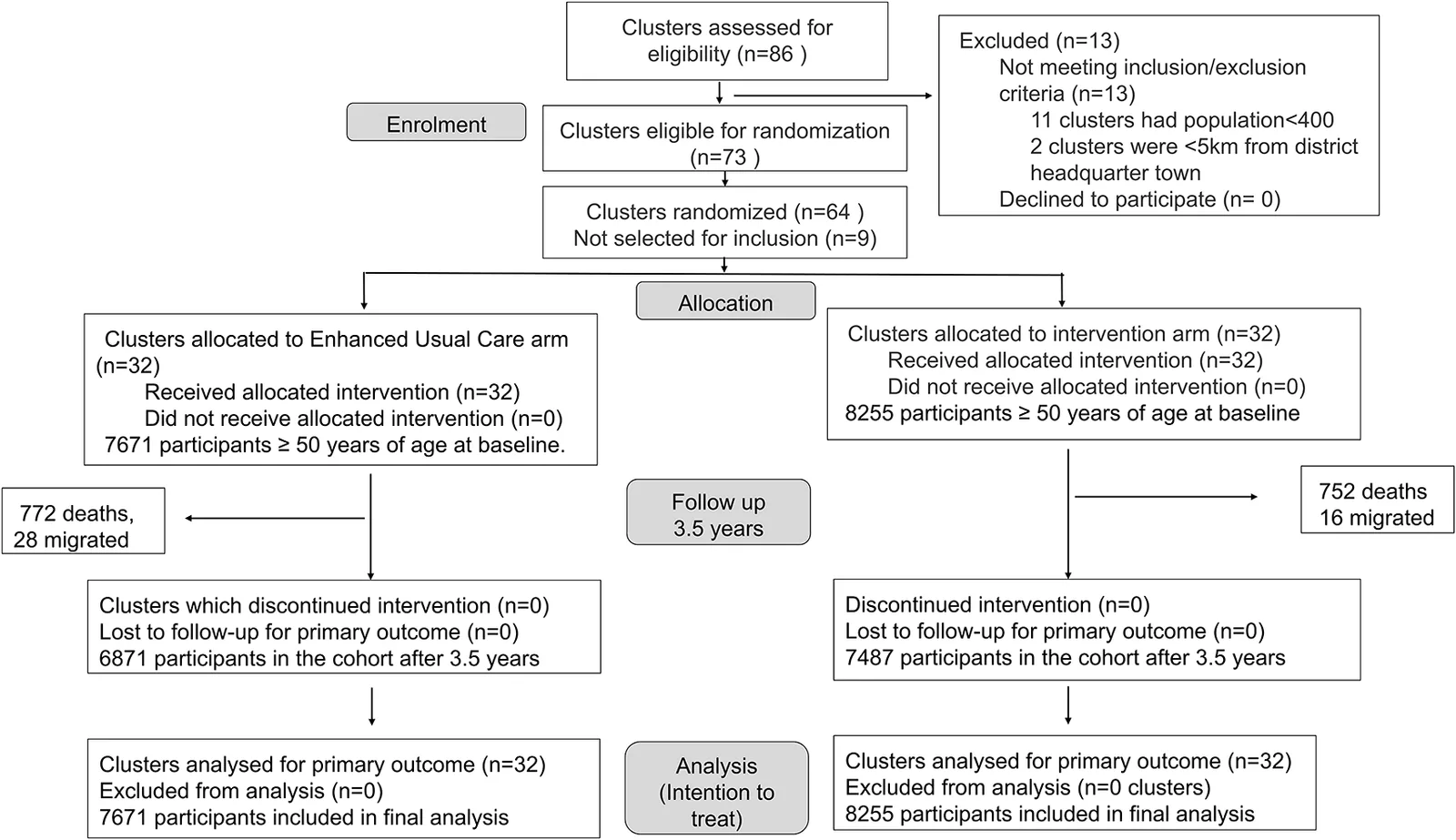
Trial profile.

From the sampling frame of 86 DSS villages, 13 villages were excluded based on the inclusion and exclusion criteria for the study-11 villages had population less than 400 and 2 villages were less than 5 kilometers from the district headquarter. From the 73 eligible villages, 32 villages were randomly assigned to the intervention arm and 32 to the control arm of enhanced usual care (EUC) ( Figure 1). Randomiszation was done by a statistician using random number generator in statistical software Stata in a way that an equal number of villages from each of the three geographic administrative blocks of the district were assigned per arm. Randomization was performed before collection of baseline data. Data analyses were conducted by a different statistician. Given the nature of the intervention, blinding patients or implementers was not feasible. However, physician coders of verbal autopsies who were the primary outcome assessors were blinded to group allocation and were unaware whether the deceased person belonged to intervention or the control arm.

### Procedures

Before implementing the intervention, community meetings were conducted in intervention villages to inform the community members regarding the nature of the intervention. The recruitment period of the study was from 01/06/2016 to 31/12/2019. This period of 3.5 years was determined based on the duration of funding and that it was a reasonable length of time to detect reduction in stroke mortality in a community setting based on a prior study. ^21^

The intervention included four key components- screening, treatment, active follow-up and awareness generation. All the human resource deployed in the intervention were recruited under the trial. Screening was conducted at baseline by newly recruited and trained (Supplementary Tables S1, S2) community health workers (CHWs) specifically for this trial as such services were not available in the public health system in this area when the trial commenced. These CHWs conducted home visits to screen all individuals aged ≥50 years in intervention villages for hypertension, diabetes, and stroke. The CHWs measured blood pressure (BP) using an electronic monitor (Omron HEM-7121) after the individual was seated for 5 minutes. If initial systolic BP (SBP) was ≥140 mmHg and/or diastolic BP (DBP) was ≥90 mmHg, a second reading was taken after 5 minutes, and the average of both readings was recorded as the BP of the individual. Urine glucose was assessed using dipsticks (Uristix, Siemens) while weight, height, waist, and hip circumference were measured using standard procedures. Stroke screening was performed using a validated questionnaire. ^22^ Individuals with BP ≥140/90 mmHg, glucosuria, or suspected stroke were referred to a mobile outreach clinic consisting of an outreach physician (OP), a pharmacist, and a driver, visiting each village every 2–4 months. The OP rechecked BP of those suspected of having hypertension, checked random capillary blood glucose (RCBG) using a glucometer (Accu-Check Active) for those with glucosuria and clinically confirmed the diagnosis of stroke. The OP confirmed diagnoses using the following criteria - a. Hypertension: BP ≥140/90 mm Hg in the outreach clinic or use of antihypertensive medications, b. Diabetes: RCBG ≥200 mg/dL in the outreach clinic or outside records showing fasting plasma glucose ≥ 126 mg/dL or a single random venous blood glucose≥ 200 mg/dL or previous diagnosis of diabetes and the use of medications for it, and c. Stroke: focal (or at times global) neurological impairment of sudden onset, and lasting > 24 hours, and of presumed vascular origin. ^23^ Patient registries were created in each intervention village for active follow up as well as treatment. The OP used a formulary of generic medicines to treat these three conditions (Supplementary Tables S3, S4, Figures S1, S2, S3). Medications were dispensed by the pharmacist after counseling, and a plastic storage box was provided to keep medicines securely. The OP treated hypertension to keep BP <140/90 mm Hg among those with hypertension and <130/80 mm Hg among those with diabetes and diagnosed chronic renal failure but to keep it > 100/60 mm Hg. Among patients with diabetes, the target was to keep RCBG between 100-200 mg/dL. Patients with clinically diagnosed stroke received atorvastatin and aspirin as per the study protocol (supplementary Figure S3). Stroke patients who had loss of consciousness or seizures at the onset of symptoms were presumed to have had a haemorrhagic stroke and were not given aspirin. All medications, urine and blood glucose testing, and storage boxes were provided free of charge. Patients with acute stroke were referred to the district hospital in the public health system where the only facility in the district for brain imaging was available or to the hospital of the host organization. The district only had services to manage stroke conservatively and acute thrombolysis or neurosurgical services were not available in the district. The patients with acute stroke were free to seek care from a secondary care facility of their choice in or outside the district. Patients had the autonomy to withdraw from the trial, discontinue treatment at any time and seek care from other providers.

CHWs conducted active patient follow up through monthly home visits to assess medication adherence, side effects, and BP control (<140/90 mmHg). They also provided information on harmful effects of tobacco/alcohol and excessive salt intake and advice for tobacco/alcohol cessation and to reduce salt intake. A curriculum was created to increase awareness among patients and communities and CHWs used videos/animations in local language for awareness generation specifically created under the trial. (e.g. https://www.youtube.com/watch?v=rcDy8AJzBrw&t=129s). During the home visit, if BP was very high (≥180/110) or on the lower side (≤99/59) or medications were poorly tolerated, CHWs consulted the OP via phone for treatment adjustments and dispensed revised prescriptions from the available stock on the same day. Both the CHWs and the OP documented these changes. If a patient missed the outreach clinic visit, the CHW supplied previously prescribed medications to ensure continuity. Medication compliance was documented by the CHWs during their home visits. It was defined as patients taking medicines exactly as prescribed by the OP over past 3 days from the day of assessment. Two field supervisors oversaw the work of 39 CHWs by visiting each CHW every 15 days and conducted annual community awareness sessions, which were open to all villagers. Various quality control measures were taken as described previously. ^16^ The risk of contamination was minimal as the villages were separated by natural boundaries and the CHWs were provided with a list of village residents from the population register and only these individuals were eligible to receive the intervention.

In the control arm, all households received informational pamphlets on tobacco cessation at the start of the intervention, given the high prevalence of tobacco use in the district. ^24^ Those in this arm could access providers at the primary health centre, block and district level hospitals in the public health system as well as private providers at the block and district headquarters for management of hypertension, diabetes or secondary prevention of stroke. Population-based screening for hypertension and diabetes gradually and variably became available in the control villages in the second half of 2018 through the NP- NCD. There was no active follow up mechanism for hypertension, diabetes or stroke in these villages, individual counseling for medication compliance or tobacco, alcohol or salt reduction or community-based awareness sessions. Treatment for these conditions was also not available within the village except for the villages which had health and wellness centres or primary health centres.

### Adverse events

We used standard medications for treatment. The adverse events were monitored by the OP, the field supervisors and the CHWs using standard data collection formats.

### Outcomes

The primary outcome of the trial was a reduction in stroke mortality in the intervention arm during the final 2.5 years of the intervention period in the cohort of individuals ≥ 50 years of age. We surmised that it would take one year to fully operationalize the intervention and thus the benefit of this preventive intervention would be seen in the later part (final 2.5 years) of the intervention. Cause of death was determined using Verbal autopsies (VA) as described in detail previously. ^4^ Briefly, VAs were conducted on all deaths by the trained field workers who were independent of the implementation team. Each VA was independently assessed by two trained physician coders who assigned cause of death using the Tenth edition of the International Classification of Disease (ICD-10) code. The physician used the method developed by the Registrar General of India for the Sample Registration System for coding the cause of death. ^25^ If the two coders did not agree, the coders were provided the disease code assigned by the other coder and were asked to reconcile the cause of death. If disagreement persisted, the cause of death was adjudicated by a third coder. Previous studies have demonstrated that VAs have high sensitivity (≥75%) and specificity (>90%) for diagnosing stroke, making them reliable for estimating stroke mortality in resource-limited settings. ^6, 26, 27^ VAs were coded independently by two trained physician coders. In cases of disagreement, a third senior physician adjudicated the cause of death. ^4^ Stroke deaths were defined according to the WHO definition, ^23^ with ICD-10 codes I64–I69 classified as stroke deaths ^4^ and I00–I99 as cardiovascular deaths.

The secondary outcomes included reduction in cardiovascular and all-cause mortality in the cohort of individuals ≥50 years of age in the intervention area in the final 2.5 years of the intervention period, the proportion of hypertensive patients on antihypertensive medication, reduction in blood pressure, proportion of diabetic patients with RCBG ≤200 mg/dL and awareness about stroke and its risk factors after the entire intervention period of 3.5 years. Non-mortality secondary outcomes were assessed via random sample surveys conducted at baseline and post-intervention on individuals ≥50 years in both arms. Individuals were drawn using simple random sampling from the list of all the individuals in this age group from the population register of the demographic surveillance system in both the arms. The samples at baseline and after the intervention were drawn independently. Field surveyors, unaffiliated with the trial implementation, conducted these surveys.

### Statistical analysis

Sample size was calculated based on the primary outcome—stroke mortality reduction. We hypothesized a 40% reduction in stroke mortality, based on an observational community-based study from Taiwan. ^21^ Using the Hayes and Bennett formula, ^28^ assuming between-cluster correlation coefficient of variation (k = 0.2) and a baseline crude stroke mortality rate of 688/100,000 (from our prior study on stroke mortality in the DSS ^4^), we required 27 villages per arm to achieve 80% power at a 5% significance level. Adjusting for 25% non-response, the final sample size was 32 villages per arm.

We calculated sample size for the random sample surveys considering 40% hypertension prevalence among individuals ≥50 years. Assuming BP control rates of 65% (intervention) vs 35% (control) post-intervention, with 80% power, 5% significance, design effect of 1.6, and 20% non-response, the required sample size was 330 individuals per arm.

The intervention’s impact was analyzed using an intention-to-treat approach with two-sided tests (α = 0.05). Both cluster- and individual-level data were used for primary and secondary outcome comparisons. The effect of the intervention on stroke, cardiovascular and all-cause mortality was assessed using Cox regression with shared frailty to account for clustering. Those lost to follow-up or those who died of a cause other than stroke or cardiovascular diseases were censored in these models. Adjustment was done for age, sex, occupation, socioeconomic status, education, and baseline stroke, cardiovascular or all-cause mortality and unadjusted and adjusted hazard ratios with 95% confidence intervals were calculated. Data for baseline stroke, cardiovascular diseases and all-cause mortality was obtained for 2.5 years prior to the intervention from the demographic surveillance system. Differences between non-mortality secondary outcomes were analysed with mixed effects linear or logistic regression models to account for clustering and adjustments were done for age, sex, education and occupation. Data were analyzed using Stata (Version 14, College Station, TX, USA) and R (R: A Language and Environment for Statistical Computing, Version 4.0.3). Minimal data were missing and no specific adjustments were made.

### Ethics approval and study registration

The study was approved by the Institutional Ethical Committee (IEC) of Society for Education Action and Research in Community Health (IEC/2014/0628-1) and the data and safety were monitored by an external Data and Safety Monitoring Committee as well as the IEC. Witnessed written informed consent was obtained for participation in the screening and intervention parts of the study and use of data without revealing personal details. Consent was obtained by the CHWs during screening and by the OP before starting treatment under the intervention **.** Participants were also informed about their freedom to quit at any stage of data collection. Confidentiality of all information obtained was maintained. The consent procedures were in accordance with the Helsinki Declaration of 1975, as revised in 2000. The trial was prospectively registered on 8 ^th^ December 2015 with the Clinical Trial Registry of India (Trial registration number- CTRI/2015/12/006424, URL- https://ctri.nic.in/Clinicaltrials/pmaindet2.php?EncHid=MTI5MzA=&Enc=&userName=CTRI/2015/12/006424) and annual approvals from the ethics committee were uploaded on the registry. There were no deviations from the study protocol during implementation. All pre-specified outcomes are reported in accordance with the protocol. We plan to disseminate the findings of the trial by publishing the key findings of the trial in local language through news and social media.

## Results

The intervention was delivered between 1 ^st^ June 2016 to 31 ^st^ December 2019. In the control and intervention villages, there were 7671 and 8255 eligible individuals ≥ 50 years of age respectively and after 3.5 years, there were 6871 and 7487 individuals after accounting for deaths and migration ( Figure 1).

The baseline demographic features of the randomized villages in the control and intervention arms as well as among the cohort of individuals ≥ 50 years of age were comparable ( Table 1). Mean age of the individuals in both the cohorts was 61 years and the proportion of women was slightly higher than men. Two thirds of individuals in both the arms were illiterate (control arm- 68.8% and intervention arm- 66.2%).

**Table 1.** Baseline demographic characteristics of the villages and the cohort of individuals ≥50 year of age in the enhanced usual care and intervention arms.

	Control arm	Intervention arm
**Characteristics of the villages** ^**#**^		
Number of villages	32	32
Total population	34883	37652
Village population	1090.1 (854.4, 1325.8)	1176.6 (962.3, 1390.9)
Population with age ≥50 years	22.1% (20.8, 23.4)	22.7% (21.8, 23.7)
Population of women among those ≥50 years of age	51.2% (50.1, 52.3)	52.3% (54.4, 53.1)
Distance from the district headquarter, km	31.0 (24.6, 37.4)	31.5 (24.5, 38.5)
Distance from a government-run Primary Health Centre, km	5.3 (3.3, 7.3)	4.8 (2.8, 6.8)
**Characteristics of the study cohort of individuals ≥50 years of age** ^**#**^		
Total population	7671	8255
**Age**		
Mean age, years	60.9 (60.4, 61.4)	61.2 (60.8, 61.6)
50-65 years	74.4% (72.4,76.4)	71.9% (69.8,73.9)
>65 years	25.6% (23.6,27.6)	28.1% (26.1,30.2)
**Sex**		
Female	51.3% (50.2,52.3)	52.7% (51.8,53.6)
Male	48.7% (47.7,49.8)	47.3% (46.4,48.2)
**Education**		
Illiterate	68.8% (64.7,72.9)	66.2% (62.7,69.6)
Literate	31.2% (27.1,35.3)	33.8% (30.4,37.3)
**Occupation**		
Farming/farm labour	96.2% (94.5,98)	95.3% (93.5,97.1)
Other	3.8% (2,5.5)	4.7% (2.9,6.5)
**Household assets**		
Families that own farmland	80.4% (76.4, 84.5)	81.7% (78.5, 84.9)
Farm land/family in acres	1.9 (1.8, 2.1)	2.1 (1.9, 2.2)
Households with a mobile phone	58.7% (53.7, 63.6)	58.1% (54.5, 61.6)
Households with television	38.3% (34.3, 42.3)	40.2% (36.7, 43.7)
Have motor bike/scooter	15.3% (13.5, 17.1)	13.3% (11.1, 15.4)

**Footnotes:** Data are n, mean (95% CI) or % (95%CI) and are mean of 32 cluster-specific values.

^#^ Data based on the population census conducted under the Demographic Surveillance System in 2015.

Access to health care, health care seeking for hypertension, diabetes and stroke and distribution of selected risk factors was assessed through the random sample surveys. The demographic features of the surveyed sample such as age, sex distribution, and education were comparable to the population in these villages which can be seen from the data in Tables 1 and demographic features of the surveyed sample in Table 2. As the cohort aged after the intervention, the mean age of the surveyed individuals increased in the sample survey at the end of the intervention. At baseline, 59.8% (196/328) and 52.3% (162/308) participants reported having their blood pressure ever checked and 20.4% (67/328) and 17.8% (55/308) reported having their blood glucose checked before the survey in the control and intervention arms respectively. 33.5% (110/328) participants in the control arm and 29.5% (91/308) in the intervention reported being diagnosed with hypertension, diabetes or stroke before the survey. Among those with self-reported diagnosis of these three conditions, only 55.5% (61/110) in the control arm and 46.2% (42/91) in the intervention arm reported taking regular treatment. Tobacco and alcohol use, body mass index and waist-hip ratio were comparable in the two arms at baseline ( Table 2).

**Table 2.** Differences in the risk factor distribution in the control and intervention arms in the sample surveys.

	Baseline		After 3.5 years	
	Control	Intervention	Control	Intervention
	n	n	n	n
**Individuals surveyed/Sample size**	328/330 (99.4%)	308/330 (93.3%)	312/330 (94.5%)	303/330 (91.8%)
**Sex**				
Females	164 (50%)	160 (51.9%)	167 (53.5%)	155 (51.2%)
Males	164 (50%)	148 (48.1%)	145 (46.5%)	148 (48.8%)
**Mean age (SD)**	61.6 (8.3)	62.5 (9)	63.4 (7.5)	64.9 (7.3) *
**Education**				
Illiterate	215 (65.5%)	198 (64.3%)	206 (66%)	192(63.4%)
Literate	113 (34.5%)	110 (35.7%)	106 (34%)	111 (36.6%)
**Occupation**				
Farmer or farm labourer	197 (60.1%)	168 (54.5%)	223 (71.5%)	193 (63.7%)
Home maker	32 (9.8%)	25 (8.1%)	21 (6.7%)	36 (11.9%)
Retired	88 (26.8%)	96 (31.2%)	58 (18.6%)	56 (18.5%)
Other (e.g. business, service)	11 (3.4%)	19 (6.2%)	10 (3.2%)	18 (5.9%)
**Ever checked blood pressure**	196 (59.8%)	162 (52.4%) ^§^	253 (81.1%)	267 (88.1%) ^§§^
**Ever checked blood glucose**	67 (20.4%)	55 (17.8%)	144 (46.1%)	169 (55.8%)
**Self-reported prior history of**				
Hypertension	96 (29.3%)	80 (26%)	97 (31.1%)	116 (38.3%)
Diabetes	14 (4.3%)	17 (5.5%)	14 (4.5%)	26 (8.6%)
Stroke	14 (4.3%)	8 (2.6%)	11 (3.5%)	14 (4.6%)
Any one of the above	110 (33.5%)	91 (29.5%)	103 (33%)	128 (42.2%)
**Treatment provider among those with self-reported hypertension, diabetes or stroke**				
Village-level provider ^#^	14 (12.7%)	12 (13.2%)	28 (27.2%)	2 (1.6%) ^**€**^
Government-run Primary Health Centre	9 (8.2%)	11 (12.1%)	25 (24.3%)	4 (3.1%) ^**£**^
Government-run District Hospital	4 (3.6%)	4 (4.4%)	4 (3.9%)	1 (0.8%)
Private practitioners	10 (9.1%)	7 (7.7%)	14 (13.6%)	9 (7%)
Outreach clinic in the intervention	0 (0%)	0 (0%)	0 (0%)	97 (75.8%)
Others (providers from alternative systems of care)	26 (23.6%)	12 (13.2%)	5 (4.9%)	2 (1.6%)
No treatment	48 (43.6%)	46 (50.5%)	31 (30.1%)	13 (10.2%) ^**¥**^
**% of patients with self-reported hypertension, diabetes or stroke taking treatment regularly** ^##^				
Hypertension	54 (56.3%)	38 (47.5%)	65 (67%)	109 (94%) ^**$**^
Diabetes	8 (57.1%)	6 (35.3%)	9 (64.3%)	18 (69.2%)
Stroke	3 (21.4%)	2 (25%)	8 (72.7%)	5 (35.7%)
Any one of the above	61 (55.5%)	42 (46.2%)	68 (66%)	113 (88.3%) ^**$**^
**Current tobacco use** ^**$$**^	239 (72.9%)	224 (72.7%)	242 (77.6%)	221 (72.9%)
Smokeless tobacco	236 (98.7%)	218 (97.3%)	237 (97.9%)	218 (98.6%)
Smoked tobacco	14 (5.9%)	16 (7.1%)	13 (5.4%)	8 (3.6%)
**Current alcohol use** ¶	68 (20.7%)	70 (22.7%)	79 (25.3%)	76 (25.1%)
**Measurements**				
Height (cm)	153.7 (8.3)	152.6 (8.6)	153.2 (8.1)	152.7 (9.1)
Weight (kg)	46.7 (9.6)	45.2 (10.2)	46.7 (9.3)	46.5 (10.5)
BMI (kg/m2)	19.7 (3.2)	19.3 (3.3)	19.8 (3.2)	19.9 (3.8)
Waist circumference (cm)	72.4 (9.7)	71.7 (10.2)	73.6 (10.8)	73.7 (10.9)
Hip circumference (cm)	83.3 (6.6)	83.2 (6.9)	83.6 (8.4)	82.6 (6.9)
Waist hip ratio	0.9 (0.07)	0.9 (0.07)	0.9 (0.1)	0.9 (0.1)

Footnotes: Data are n (%) or mean (SD)

P-values were calculated using a mixed effects linear or logistic regression models to account for clustering. Adjustments were done for age, sex, education and occupation. Only significant p-values are highlighted.

^#^ often unskilled or semi-skilled, unlicensed informal providers

^##^ regular use was defined as taking prescribed medicines daily for 3 consecutive days before the survey, the total of patients taking medicines for any of the above conditions is smaller than the sum of those with individual condition as some patients had more than one conditions.

^$$^ self-reported current use of tobacco. % of smokeless and smoked tobacco users is out of total tobacco users. As some individuals used both smokeless and smoked tobacco products the total of the two is >100%

^¶^ any alcohol use within the last 6 months

§ p = 0.04,

^§§^ p = 0.024,

^€^ p < 0.001,

^£^ p < 0.001,

^¥^ p = 0.003,

^$^ p = 0.001.

^*^ Unadjusted p = 0.027.

**Figure 2.**
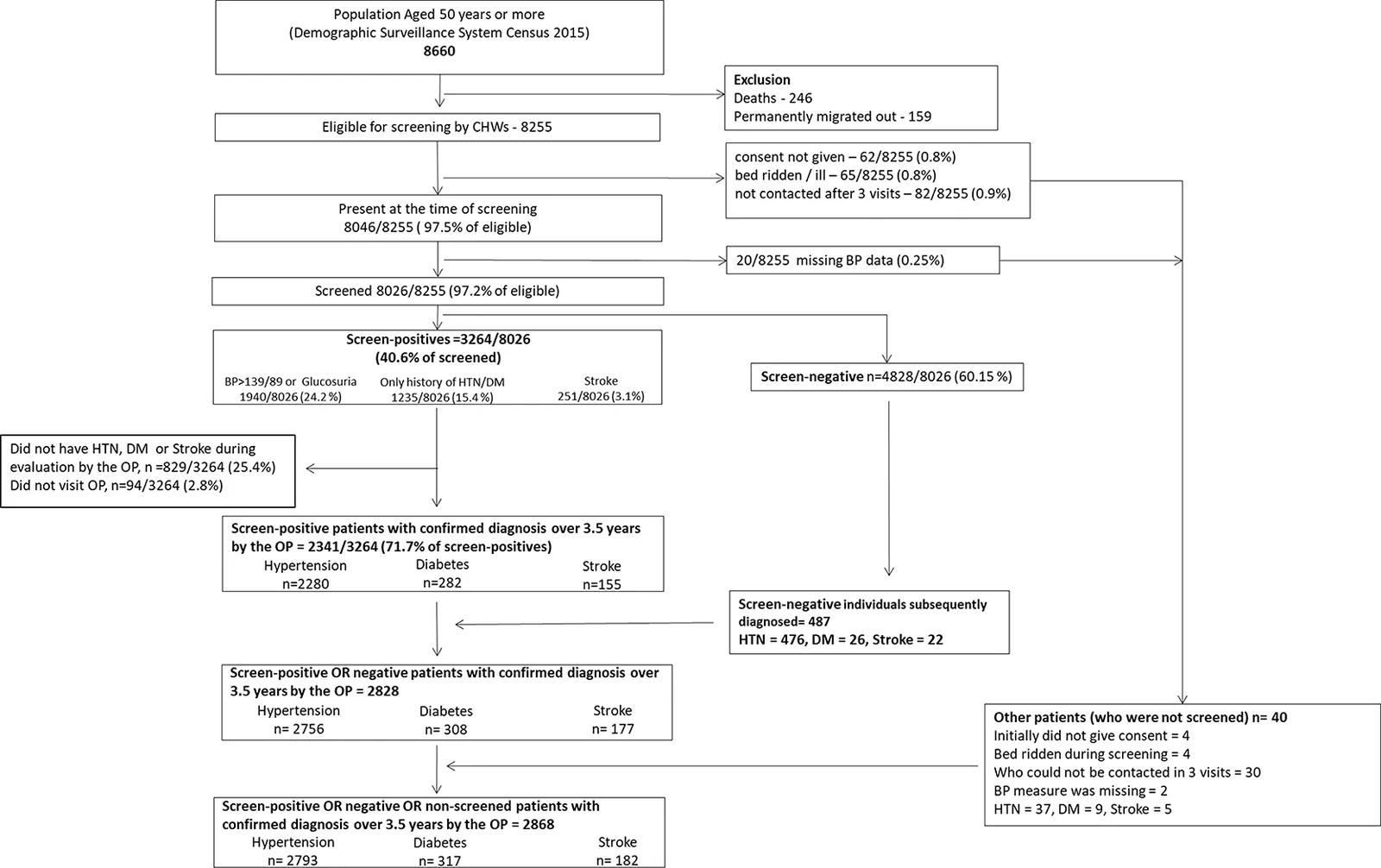
Flow diagram for screening, diagnosis and treatment of patients with hypertension, diabetes or stroke in the intervention arm.

**Figure 3.**
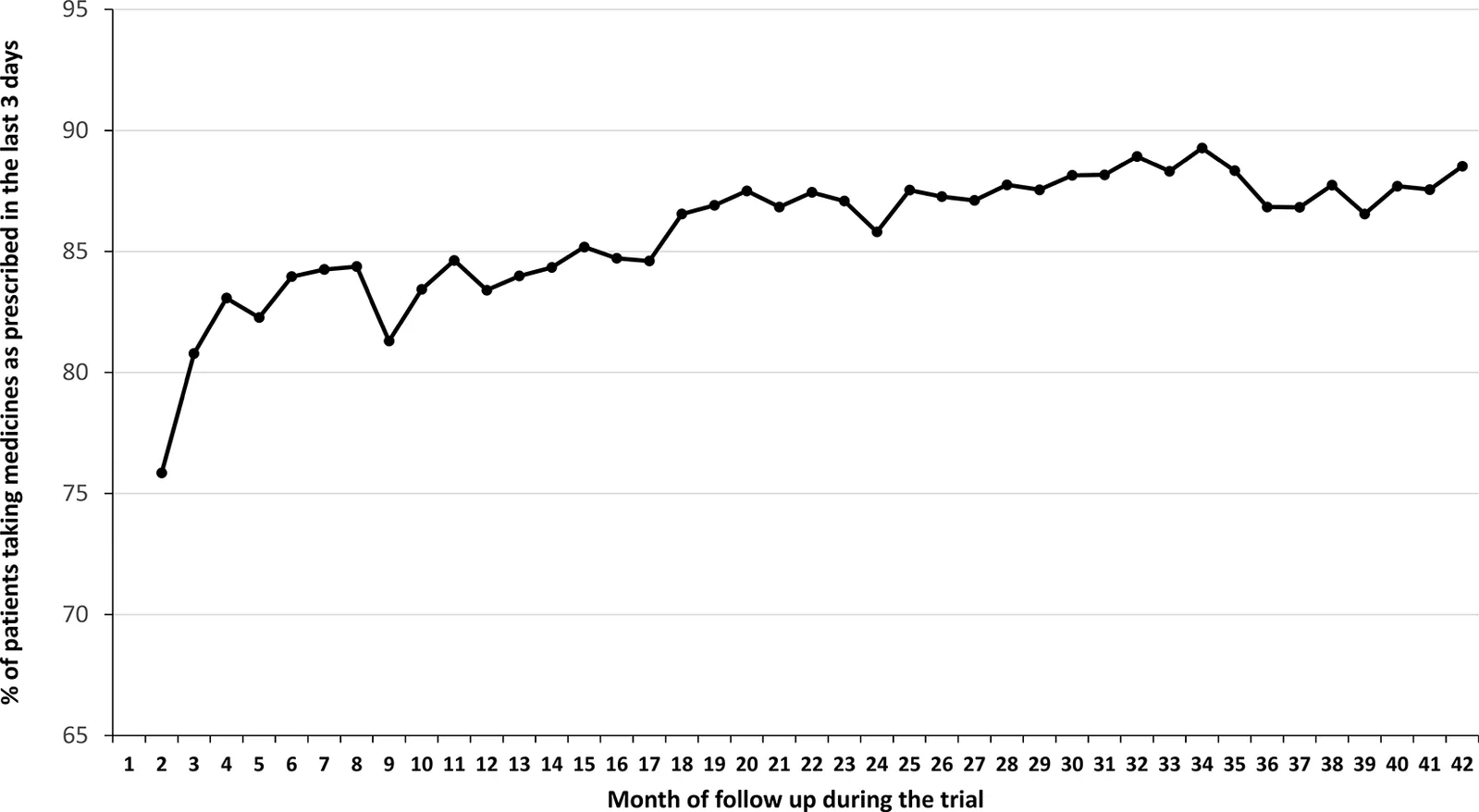
Compliance with trial medications (taking medications as prescribed by the outreach physician in the past 3 days) as assessed during home visits by the community health workers.

In the intervention arm 8026 (97.2%) out of 8255 eligible individuals ≥50 years of age were screened. Among these 2341 (28.4%) were diagnosed with one of the three conditions ( Figure 2). Over the intervention period of 3.5 years, out of 8255 eligible individuals, 2793 (33.8%) were diagnosed with hypertension, 317 (3.8%) with diabetes and 182 (2.2%) with stroke and received treatment. Visiting the villages every 2-4 months, the OP made 16 visits to the intervention villages during the intervention period and evaluated 83.5% of the eligible patients on average during these visits (Supplementary Figure S4). After the screening, the CHWs visited 90.7% of the eligible patients on average for monthly follow-ups during the intervention period (Supplementary Figure S5). The compliance of patients to the medications prescribed in the intervention improved over time ( Figure 3) and was between 85-90% in the last months of the intervention.

After the intervention period, access to blood pressure and blood glucose measurement increased in both the arms with more than 80% reporting their blood pressure and close to 50% reporting their blood glucose being ever checked ( Table 2). This access was more in the intervention arm with 7% (95% confidence interval [1.4,12.7%], p = 0.024) more individuals reporting blood pressure and 9.6% (95% confidence interval [1.8,17.5%], p = 0.055) more reporting blood glucose measurements in the cross-survey at the end of the intervention. The percentage of individuals self-reporting to have hypertension, diabetes or stroke was higher in the intervention arm compared to the control arm. In the intervention arm, 75.8% (97/128) reported taking treatment from the outreach clinic ( Table 2, supplementary Table S5).

At baseline, the number of hypertensive patients taking regular medicines was comparable in the control and the intervention arm (56.3% vs 47.5%, p = 0.22). After the intervention this percentage increased to 67% (65/97) and 94% (109/116) respectively (p = 0.001) respectively.

The mean SBP and DBP in the control (130.1 and 82.8 mmHg,) and the intervention arm (131.5 and 82.7 mmHg) were comparable at baseline. After the intervention, as the cohort aged, these increased in both the arms. The mean SBP was 135.7 mmHg in the control arm and 132.1mm Hg in the intervention arm, being lower by 3.6mmHg in the latter (p = 0.016). The mean DBP was 84.8 mmHg in the control arm and 82.4 mmHg in the intervention arm, being lower by 2.3mmHg in the intervention arm (p = 0.017). Among patients with self-reported hypertension, after the intervention, the mean SBP was lower by 8.1mm Hg (p = 0.001) and mean DBP by 5.1mmHg (p = 0.003) in the intervention arm compared to the control arm ( Table 3, supplementary Table S6). (p = 0.003) in the intervention arm compared to the control arm ( Table 3, supplementary Table S6).

**Table 3.** Effect of the intervention on hypertension, blood glucose control and awareness about stroke in the sample surveys.

	Baseline		After 3.5 years	
	Control	Intervention	Control	Intervention
**Hypertension**				
Among all surveyed individuals-				
SBP, mmHg	130.1 (127.8,132.4)	131.5 (129.2,133.8)	135.7 (133.3,138)	132.1 (130.1,134.1) ^§§^
DBP, mmHg	82.8 (81.5,84.1)	82.7 (81.5,84)	84.8 (83.5,86)	82.4 (81.3,83.5) ^€^
Controlled blood pressure (<140/90)	217/328 (66.2%)	203/308 (65.9%)	185/312 (59.3%)	203/303 (67%) ^**£**^
Among patients with self-reported hypertension-				
SBP, mmHg	139 (133.9,144)	141.4 (136.7,146.1)	144 (139.9,148.1)	135.9 (133.1,138.7) ^¥^
DBP, mmHg	86.1 (83.3,88.8)	85.5 (82.9,88.1)	88.1 (85.8,90.5)	83 (81.5,84.5) ^$^
Controlled blood pressure (<140/90)	49/96 (51%)	39/80 (48.8%)	42/97 (43.3%)	71/116 (61.2%) ^**$$**^
**Diabetes**				
Among all surveyed individuals-				
RCBG (mg/dL), 95% CI	115.4 (111.5,119.3)	118.0 (113.6,122.4)	113.4 (109.3,117.5)	119.4 (114.2,124.5)
Individuals with RCBG <200 mg/dL	318/328 (97%)	298/308 (96.8%)	301/312 (96.5%)	291/303 (96%)
Among patients with self-reported diabetes-				
RCBG (mg/dL)	174.8 (117.3,232.3)	169.9 (121.5,218.4)	172.7 (127.9,217.6)	164.7 (123.3,206.1)
Patients with RCBG <200 mg/dL	9/14 (64.3%)	11/17 (64.7%)	9/14 (64.3%)	20/26 (76.9%)
**Awareness about stroke**				
Among all surveyed individuals-				
Correct response to “Which body organ is affected in stroke?”	3/328 (0.9%)	2/308 (0.6%)	14/312 (4.5%)	61/308 (20.1%) ^*^
Correct response to “What should be done to prevent stroke?”	38/328 (11.6%)	18/308 (5.8%)	45/312 (14.4%)	86/308 (28.4%) ^*^
Correct response to any of the above	40/328 (12.2%)	20/308 (6.5%)	54/312 (17.3%)	108/303 (35.6%) ^*^
Among patients with self-reported hypertension, diabetes or stroke				
Correct response to “Which body organ is affected in stroke?”	3/110 (2.7%)	1/91 (1.1%)	6/103 (5.8%)	50/128 (39.1%) ^*^
Correct response to “What should be done to prevent stroke?”	16/110 (14.6%)	9/91 (9.9%)	17/103 (16.5%)	58/128 (45.3%) ^*^
Correct response to any of the above	18/110 (16.4%)	10/91 (11.0%)	20/103 (19.4%)	73/128 (57.0%) ^*^
**Salt use- taking added salt during meals**				
Among all surveyed individuals-	NA	NA	153/312 (49%)	124/303 (40%)
Among patients with self-reported hypertension, diabetes or stroke	NA	NA	43/103, (41.7%)	32/128 (24.2%) ^**^

Footnotes: Data are n (%) or mean (95%CI)

SBP- systolic blood pressure, DBP- diastolic blood pressure, RCBG- random capillary blood glucose, NA-not assessed.

P-values were calculated using mixed-effects linear or logistic regression models to account for clustering. Adjustments were made for age, sex, education and occupation. Only significant p-values are highlighted.

^§§^ p = 0.016,

^€^ p = 0.017,

^£^ p = 0.042,

^¥^ p = 0.001,

^$^ p = 0.003,

^$$^ p = 0.021,

* p = <0.001,

** p = 0.00.

The mean RCBG levels and the percentage of individuals with RCBG <200 mg/dL were comparable at baseline and after the intervention in the two arms as well as among those with. self-reported diabetes.

The awareness about stroke increased in the intervention arm after the intervention with 18.3% (p < 0.001) more individuals providing correct response to either of the following questions “which body organ is affected in stroke?” or “what should be done to prevent stroke?.”

The intervention had no effect on alcohol or tobacco use ( Table 2, Supplementary Table S5). Among those with self-reported hypertension, diabetes or stroke, lower number of patients in the intervention arm reported taking added salt in diet compared to those in the control arm (24.2% vs 41.7%, p = 0.005) after the intervention.

**Figure 4.**
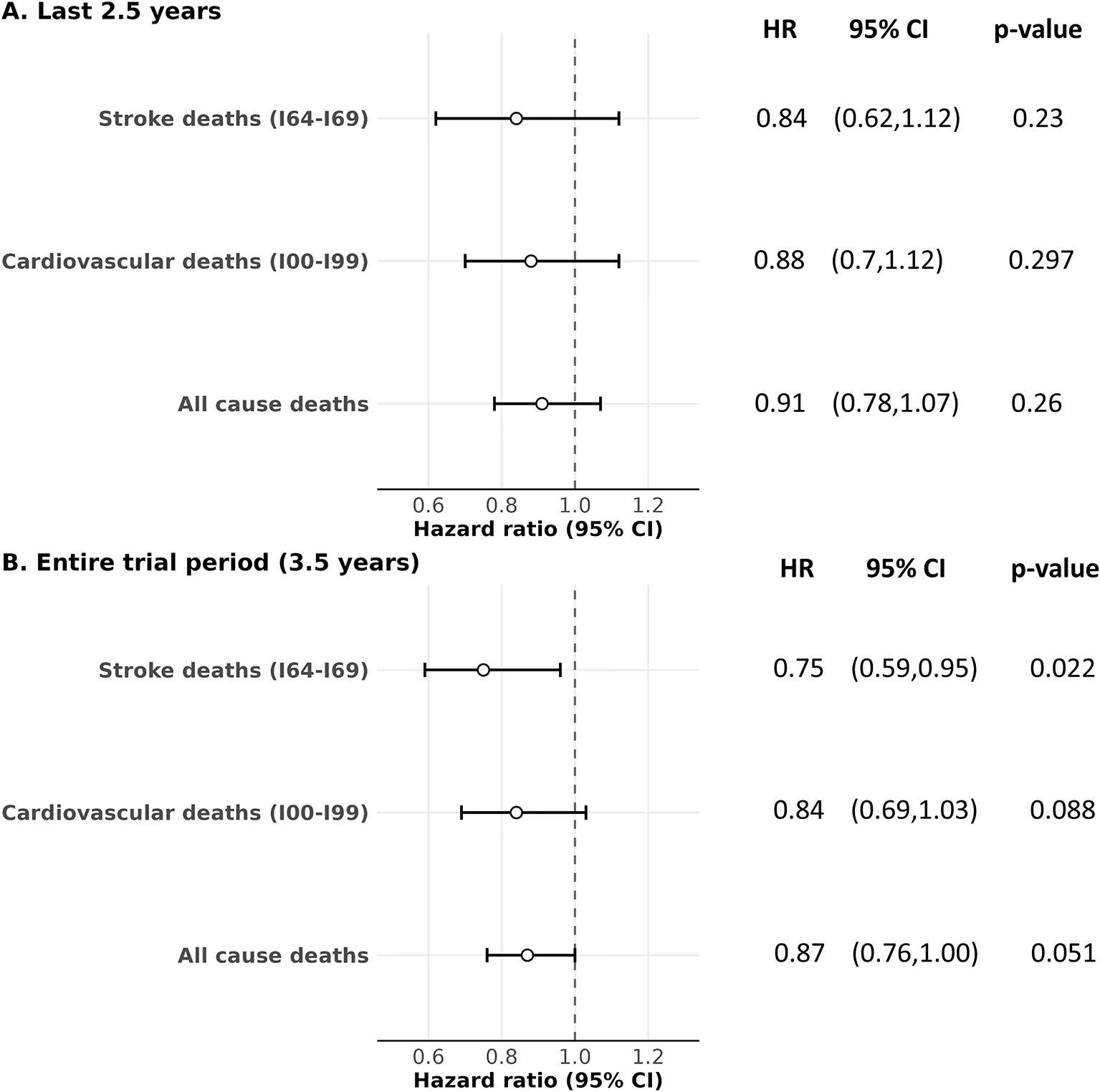
Forest plot of hazard ratios (HRs) showing the effect of the intervention on stroke, cardiovascular and all-cause mortality A. Intervention versus control in the last 2.5 years of the intervention, and B. Intervention versus control over the entire trial period of 3.5 years (post-hoc analysis).

Cause of death on all deaths was determined using verbal autopsies. The information on the causes of death in both the arms at baseline, over the last 2.5 years as well as the entire trial period and the agreement between the verbal autopsy coders is provided in supplementary Tables S7 and S8 respectively. At baseline, the stroke mortality rate per 100,000 person-years was 552.3 and 582.2 in the control and intervention arms respectively ( Figure 3 and Supplementary Table S9). We did not have data on the cause of death among individuals who had permanently migrated. At baseline, the stroke mortality rate per 100,000 person-years was 552.3 and 582.2 in the control and intervention arms respectively ( Figure 4 and Supplementary Table S9). There was no reduction in stroke mortality rate in the last 2.5 years of the intervention (adjusted hazard ratio 0.84, 95% confidence interval [0.62,1.12], p = 0.23). In a post-hoc analysis, it was reduced by 25% in the intervention arm compared to the control arm (adjusted hazard ratio 0.75, 95% confidence interval [0.59,0.95], p = 0.022) over the entire trial period of 3.5 years. There was no reduction in mortality rate due to cardiovascular diseases in the intervention arm in the last 2.5 years of the intervention (adjusted hazard ratio 0.88, 95% confidence interval [0.7,1.12], p = 0.297) as well as over the entire trial period (adjusted hazard ratio 0.84, 95% confidence interval [0.69,1.03], p = 0.088). All-cause mortality rate was also not reduced in the last 2.5 years of the intervention (adjusted hazard ratio, 0.91, 95% confidence interval [0.78,1.07], p = 0.26) in the intervention arm and over the entire trial period in a post-hoc analysis (adjusted hazard ratio, 0.87, 95% confidence interval [0.76,1.00], p = 0.051).

Adverse events related to the use of trial medications were mostly mild (Supplementary Tables S10 – S14) and resolved when the medications were changed. Serious adverse events possibly or definitely related to the medications used in the trial were recorded in 45 (1.6%) patients over the entire duration of the trial ( Table 4).

**Table 4.** Serious adverse events among patients receiving the trial intervention over the entire follow up period of 3.5 years.

	n	% out of total patients (n = 2868)
**Total SAEs**	87	3.0
*Pre-defined events* *		
Any hospitalization	28	0.9
Acute myocardial infarction	2	0.1
Stroke	2	0.1
Heart failure	2	0.1
Renal failure	1	0.0
**SAEs possibly or definitely related to the trial medications**	45	1.6
*Pre-defined events* *		
Clinically suspected hypotension/syncope	16	0.6
Hypotension-documented	1	0.0
Clinically suspected hypoglycaemia	7	0.2
Hypoglycaemia-documented	3	0.1
Any hospitalization	7	0.2
Fall with injury needing evaluation in hospital with or without hospitalization	4	0.1
Generalised weakness	1	0.0

* Events can be overlapping.

## Discussion

The results of this trial show that a context-sensitive, multicomponent intervention involving community health workers and addressing local barriers to health care for hypertension, diabetes and secondary prevention of stroke was acceptable and led to improved access to care, blood pressure control and awareness about stroke among those ≥50 years of age. However, while there was a reduction in stroke mortality over the last 2.5 years of the intervention, which was the primary outcome of the trial, this reduction did not achieve statistical significance. Our study adds to the very limited literature on community-based interventions for reducing stroke mortality in a resource-limited rural area.

With rapid epidemiological transition the number of patients with hypertension, diabetes and stroke is rising fast in India. There were an estimated 207 million persons with hypertension, 77 million with diabetes and 9.6 million with stroke in India. ^29– 31^ A significant proportion of these patients live in rural areas where access to care for NCDs is limited. Given the challenges to health care access, interest in decentralised preventive interventions for NCDs which involve community health workers has increased over the years. The community-based method that we developed could be delivered with good coverage. CHWs visited more than 90% and the OP visited more than 80% of the patients. The trial results also showed that this intervention was acceptable to people. Close to 75% of all patients with hypertension, diabetes and stroke in the intervention cohort reported taking medicines from the outreach clinic. The intervention also offered significant convenience to patients.

The access to blood pressure and blood glucose measurement increased in both the arms during the intervention period, possibly due to the population-based screening under the NP-NCD in this district, but was more in the intervention arm. The percentage of patients with hypertension who reported taking medicines regularly was also significantly higher in the intervention arm. This indicates that in addition to population-based screening, ensuring uninterrupted care to patients, active follow up by the CHWs through home visits and awareness generation led to increased acceptance and compliance with medications, the latter components are currently not included in the NP-NCD.

The SBP was reduced by 3.5mm Hg and DBP was reduced by 2.4 mm Hg in the cohort of individuals ≥50 year of age in the intervention arm indicating a reduction in blood pressure at the community-level. SBP was decreased by 8.1 mm Hg and DBP by 5.1mm Hg among those with hypertension. The reduction in SBP observed among hypertensive patients in this study is close to that reported from studies in the LMICs where there was 8-11 mm reduction in SBP) among hypertensive patients in community-based intervention involving CHWs. ^32– 35^ The reduction in blood pressure among hypertensive patients in this study was higher than that reported in a recent meta-analysis of studies involving blood pressure reduction with task sharing with CHWs from LMICs where there was a mean reduction of 3.7 mm Hg in SBP. ^36^ Our findings indicate that, the intervention could achieve a modest reduction in blood pressure at the population-level and a higher reduction among those with hypertension.

The intervention had no effect on the control of blood glucose among all surveyed individuals. Several factors could have contributed to this lack of difference between the two arms. We used urine glucose for screening and random blood glucose to assess response to treatment and evaluate the effect of the intervention both of which could be insensitive indicators. Use of point of care hemoglobin A1C and measurement of fasting and post-meal glucose by trained community health workers may help to improve monitoring of diabetes but would need training of CHWs to conduct such blood tests. Similarly, use of more than two oral hypoglycemic agents, and insulin where needed, may help to improve control of diabetes in community settings. Further research will be needed on community-based interventions which can improve blood glucose control in such under resourced rural settings.

Generating awareness in an elderly population where most individuals were illiterate was a challenging task. However, awareness about stroke and its risk factors did increase significantly after the intervention, likely due to culturally appropriate awareness material. There is a need to create such audiovisual material in various languages in India which appeals to masses and takes into consideration low levels of school education.

Tobacco and alcohol use did not change after the intervention. The findings highlight the limitation of individual counseling in controlling these risk factors in communities where their acceptance is high. Use of nicotine replacement therapy or other pharmacological agents, behavioral therapies and policy interventions to raise prices of tobacco may be needed in such settings to reduce harm due to tobacco. Similarly for reducing harmful effects of alcohol, interventions such as community mobilization, increasing local enforcement of alcohol laws, limiting access to alcohol and increasing prices may be evaluated. The percentage of individuals reporting added salt use was significantly reduced among patients with hypertension, diabetes or stroke in the intervention arm. This indicates that through individual counseling such a behavioural change could be achieved for salt reduction.

Stroke mortality was reduced over the entire trial period of 3.5 years but not over the last 2.5 years of the intervention. In a trial of a community-based intervention for management of hypertension in rural areas of South Asia, there was a reduction in all-cause deaths over a period of 24 months. ^32^ However, this assessment was done at individual level while in our study reduction in stroke and mortality were assessed at community level thus the impact of the intervention is expected to be diluted. Furthermore, the prevalence of risk factors such as obesity, diabetes was relatively low in this rural agrarian community. Our results imply that the community-based intervention will need to be implemented for at least 3.5 years to reduce stroke mortality in such a setting. For reduction in cardiovascular and all-cause mortality, periods longer than 3.5 years may be necessary.

Our results have important implications for control of cardiovascular risk factors and awareness in rural India. Under the government scheme called Ayushman Bharat programme, CHWs called Accredited Social Health Activists (ASHAs) have started population-based screening of individuals 30 years of age and above for hypertension and diabetes. A role for ASHAs in increasing community awareness and follow up of patients to improve treatment compliance is also envisaged as per the recent guidelines under the NP-NCD programme. ^37^ Health and Wellness Centres (HWCs) are being created to serve populations of about 3000-5000. Community Health Officers (CHOs) at these centres are providing treatment for hypertension and diabetes and are expected to make village visits to evaluate patients. Findings from our study suggests that periodic village visits by these providers (CHOs) along with regular active local follow-up by the ASHAs in the government-run health system, uninterrupted free supply of medicines for hypertension, diabetes and secondary prevention of stroke and availability of such medicines at village level can potentially help to improve care for these conditions. The study results provide evidence for an important role of CHWs in community-based management of NCDs. In our intervention, community health workers (CHWs) conducted monthly home visits. Similar visit schedules were used in trials from rural areas of South Africa, Kenya, and Uganda, which demonstrated significant improvements in blood pressure control. ^34, 35^ While such frequent visits may be considered difficult to sustain within public health systems, we argue that from an equity, access and safety perspective, they are particularly valuable in settings with low literacy levels and limited access to care. Regular home visits can enhance follow-up and blood pressure control, ultimately reducing cardiovascular events in the long term. With the ongoing epidemiological transition, the number of individuals living with chronic conditions is rising. In our trial, each cluster, with an average population of about 1,000, had nearly 100 individuals with hypertension, diabetes, or stroke. We therefore believe that deploying a second ASHA or a CHW per village may be necessary to manage the growing burden of non-communicable diseases (NCDs) in rural India, especially since ASHAs’ current responsibilities are primarily focused on maternal and child health and infectious diseases. From a pragmatic standpoint, until such intense interventions can be delivered, a graded approach from the current level of care may be considered. Such an approach can include availability of medications in the villages, more rigorous follow up of those with higher cardiovascular risk (e.g. BP >160/100, or those who have both hypertension and diabetes), three monthly instead of monthly follow up visits by ASHAs, engaging ANMs in addition to ASHAs in patient follow up and greater use of facility-based NP-NCD services for stable patients.

The study has several strengths. It was conducted in a well-defined population in a demographic surveillance site. As the trial was conducted in a real life setting in a rural area where access to health care is limited, the findings are likely to be generalizable to wider rural areas of India and potentially other LMICs. There are also several limitations. First, during the intervention period, screening and treatment of hypertension and diabetes started under the NP-NCDs in Gadchiroli district after 2018. The impact of this programme can be indirectly assessed from the findings of the sample surveys in the control arm. At the beginning of the intervention, about 60% and 20% of individuals reported ever checking blood pressure and blood glucose. By the end of the intervention these percentages increased to about 80% and 46% in the control arm. Similarly, 8% patients with self-reported hypertension, diabetes or stroke reported taking medicines from the government run primary health centres at baseline. This percentage increased to 24% by the end of the intervention. This increase in screening and access to care in the control arm during the intervention period, which could partly be attributed to the NP-NCD programme, could have led to reduced mortality difference in the two arms of the trial. Because NP-NCD is delivered as a package of services which included screening, referral, diagnosis and treatment and participants sought care from multiple public and private providers over time, it was not possible to reliably quantify the effect of this programme on trial results. After the trial was over, we did refer the patients in the intervention arm to the government operated health centres to ensure continued care. At the time the study was conceived, the NP-NCD had not yet been implemented in the study region, and the intervention was therefore delivered outside the public health system. While this limits immediate generalizability to settings where the programme is fully operational, it allowed us to test certain activities which are currently not a part of the NP-NCD such as active follow up and availability of medications in the village which led to high medication compliance rate. Importantly, the findings provide timely evidence to inform policy and programme design as NP-NCD is implemented across India. Second, the trial was powered to detect a reasonably large reduction (40%) in stroke mortality in the final 2.5 years of the intervention in this population where access to care was limited. It seems that the trial may have been underpowered to detect such a difference over a shorter time span of 2.5 years in real world setting. There was also an assumption that in the first year of the intervention we would not be able to observe any effect on stroke mortality. The post-hoc analysis suggests that this is not the case and benefit on stroke mortality could be accrued even within the first year of the intervention. Third, we did not assess lipid levels in individuals in the random sample surveys or among stroke patients before starting statins to assess for hypercholesterolemia. People in this region were very reluctant to give venous blood samples. Therefore, this component was excluded. However, the distribution of the other risk factors was comparable in the two arms so the likelihood that the two arms differed in terms of lipid levels is low. Also, statins offer benefit in preventing subsequent episodes of stroke irrespective of lipid levels. ^38^ Fourth, patients with stroke were given aspirin using clinical criteria to exclude hemorrhagic stroke as brain imaging was not easily available in this region. It is less likely that this approach had a detrimental effect on the patients as large studies where aspirin was given to stroke patients without brain imaging did not show increase in the risk of death. ^39, 40^ As a precautionary measure, the adverse events among patients using aspirin were systematically monitored and reported to the Data and Safety Monitoring Committee. No excess adverse events were observed among those using aspirin (Supplementary Table S12). Fifth, blood glucose control was monitored using RCBG instead of fasting blood glucose or hemoglobin A1c. This was done as people were reluctant to provide venous glucose, logistic challenge associated with collecting fasting venous blood samples in villages and lack of reliable point of care tests for HbA1c when the trial was started. Sixth, the publication of this manuscript was delayed which can influence the generalizability considering potential temporal changes. The delay was initially due to COVID-19 pandemic and then due to institutional data-sharing and contractual constraints to publication following a change in institutional affiliation of the Principal Investigator. These processes did not affect the conduct of the trial, data integrity, or prespecified analyses. Given that this research was publicly funded, the study funder encouraged dissemination, and we report the results here in accordance with principles of transparency and responsible reporting.

Blood pressure reduction, increasing awareness about stroke could be achieved with a community-based intervention involving task sharing with CHWs in an under resourced rural community where most of the participants had low levels of education. The components of this intervention such as creation of patient registries, active follow up through CHWs, periodic village visits by a physician, availability of medicines with the CHWs and facility for teleconsultation with a physician, where needed, can be incorporated in the NP-NCD to improve care of NCDs in rural India. Such an intervention can be scaled to other underdeveloped rural areas in LMICs and can play an important role to attain the Sustainable Development Goal of reduction in premature mortality from cardiovascular diseases. ^41^

## Contributors

YK designed the study and developed analysis plan with inputs from SN. SSG and YK analysed data. All other authors were involved in the implementation or data collection of the trial. YK drafted the initial version of the manuscript. All authors critically reviewed and approved the final manuscript.

## Data Availability

Open access to individual level data is restricted due to possible ethical and data security issues arising from contractual obligations. Summary statistics and data are available under the extended data. Request for additional data may be submitted in the form of a brief proposal to the Principal Investigator Yogeshwar Kalkonde at yvkalkonde@gmail.com. Repository name: Open Science Framework repository: WOR_supplementary material_stroke trial-Kalkonde et al. at **https://doi.org/10.17605/OSF.IO/7Q5TJ** . ^42^ The study contains following extended data
1. Supplementary material Supplementary Table 1. Training of providers in the trial Supplementary Table S2. Inter-rater agreement between a physician and community health workers in assessing anthropometric parameters and blood pressure. Supplementary Table S3. Formulary of trial medications Supplementary Figure S1: Hypertension treatment algorithm Supplementary Figure S2: Diabetes mellitus treatment algorithm Supplementary Table S4. Starting dose of oral hypoglycemic agents based on random capillary blood glucose levels Supplementary Figure S3: Stroke preventive treatment algorithm Supplementary Figure S4. Coverage of patients (percentage of enrolled patients being evaluated) with hypertension, diabetes and stroke in the outreach clinic Supplementary Figure S5. Coverage of the home visits by the community health workers Supplementary Table S5. Differences in the risk factor distribution in the control and intervention arms in the sample surveys Supplementary Table S6. Effect of the intervention on hypertension, blood glucose control and awareness about stroke in the sample surveys- differences between the control and intervention arms Supplementary Table S7. Causes of death in the enhanced usual care and the intervention arms of the trial Supplementary Table S8. Agreement between physician coders of verbal autopsies in coding all deaths, deaths due to all cardiovascular diseases (CVD) and stroke over the entire trial period Supplementary Table S9. Stroke, cardiovascular and all-cause mortality in the cohort of individuals ≥ 50 years of age in the enhanced usual care and the intervention arms Supplementary Table S10. Adverse drug reactions reported by patients with hypertension attending the outreach clinic over the entire follow up period of 3.5 years Supplementary Table S11. Adverse drug reactions reported by patients with diabetes mellitus attending the outreach clinic over the entire follow up period of 3.5 years Supplementary Table S12. Adverse drug reactions reported by patients with stroke attending the outreach clinic over the entire follow up period of 3.5 years Supplementary Table S13. Comparison of adverse events in the two arms of the trial using selected indicators in the random sample survey conducted at the end of the intervention. 2. Trial protocol 3. Operational definitions 4. CONSORT 2025 checklist 5. TIDieR checklist available under the Creative Commons Attribution 4.0 International (CC BY 4.0) license
