# Supplementary material for: Effect of a community-based intervention for cardiovascular risk factor control on stroke mortality in rural Gadchiroli, India: a cluster-randomised controlled trial
Source: Wellcome Open Res. 2026 Jul 8;11:209. Originally published 2026 Apr 9. [Version 2] doi: 10.12688/wellcomeopenres.26002.2 (PMC13598393; doi:10.12688/wellcomeopenres.26002.2)
Supplement: Peer review history

# Reviewer report: Reviewer response for version 2

**DOI:** 10.21956/wellcomeopenres.29934.r167292

**Published:** 2026-09-23

**Authors:** Giridhara R Babu

**Affiliations:** 1 Qatar University, Doha, Qatar

This trial was conducted in 64 villages (32/32), 1:1, stratified by 3 blocks; closed cohort aged ≥50 y (7,671 controls; 8,255 intervention); 3.5 y intervention (Jun 2016–Dec 2019); DSS verbal autopsy for all deaths. Among these villages, 97.2% were screened; 33.8% had hypertension, 3.8% had diabetes, and 2.2% had stroke treated; CHW coverage >90%, outreach physician >80%; adherence 85–90%. The primary outcome (2.5 y) was stroke mortality: 416.7 vs 484.9/100,000 py; aHR 0.84 (95% CI 0.62–1.12), p=0.23. Full 3.5 y: aHR 0.75 (0.59–0.95).

 • Other mortality: CVD aHR 0.88 (0.70–1.12), 0.84 (0.69–1.03); all-cause 0.91 (0.78–1.07), 0.87 (0.76–1.00).

 • Outcomes: SBP −3.6 / DBP −2.3 mmHg; hypertensives −8.1 / −5.1 mmHg; medication 94% vs 67%; BP control 61.2% vs 43.3%; stroke awareness +18.3%; salt intake 24.2% vs 41.7%. No effect on RCBG, tobacco, or alcohol. SAEs 1.6%.

 • Powered for a 40% reduction assuming a baseline of 688/100,000 py and k=0.2; observed baseline 552.3 (control) and 582.2 (intervention); ~170–180 stroke deaths in the primary window, roughly 80% power for a 35–40% reduction, 20% for 16%.

**Please consider including the following**

1. Hazard ratios for all three mortality outcomes are reported, and all-cause deaths appear in Figure 1 (772 control, 752 intervention; crude 10.1% vs 9.1% over 3.5 y), but the numbers of stroke and cardiovascular deaths and the person-years behind every rate sit only in Supplementary Table S9. Report these by arm and period, with crude and age-standardized rates, rate differences, unadjusted and adjusted HRs, and the per-cluster event distribution (CONSORT item).
2. Figure 2 derives 8,255 after subtracting 246 interim deaths and 159 out-migrants from the 2015 census; it is unclear whether identical enumeration date and exclusions applied to the control arm (7,671), or whether intervention follow-up began at screening. Differential left-truncation/immortal time would favor the intervention. Must be resolved.
3. State unambiguously how non-stroke CVD deaths were handled in the stroke model; add cumulative incidence (Aalen–Johansen) and Fine–Gray analyses; note that censoring on CVD death (aHR 0.84) may be informative.
4. Report the frailty distribution and variance, the implied ICC/median HR, and the observed between-cluster CV vs the assumed k=0.2. With ~2–3 events per cluster, add a cluster-level Poisson analysis and, ideally, a randomization-based permutation test.
5. The interval is compatible with a 24% reduction and excludes little, but not the null, and a 13% lower all-cause mortality in a cohort aged ≥50 y would be a large public health effect. 
6. The ordering stroke < cardiovascular < all-cause indicates a cause-specific effect diluted by non-susceptible deaths and misclassification; chance or differential death ascertainment would not produce this pattern. All-cause mortality, unaffected by cause-of-death misclassification, also moves 13%, reinforcing validity. Conversely, if stroke accounts for 12–15% of deaths and its mortality decreased 25%, the resulting all-cause reduction would be only 3–4%. An observed 13% reduction suggests a broader cardiovascular benefit, chance, or residual confounding. Please clarify which.
7. With VA sensitivity 0.75, specificity 0.90, and stroke ~12–15% of deaths, roughly half of assigned stroke deaths are false positives; a true HR of 0.75 would be observed as ≈0.85, i.e., the primary estimate is consistent with a diluted 25% effect. Consider doing another study with detailed quantitative bias analysis; report stroke and ill-defined-cause fractions and kappa by arm; state whether VA narratives were redacted of intervention cues before coding.

**Other considerations**

- Frame the NP-NCD rollout explicitly as an intercurrent event (ICH E9(R1) treatment-policy estimand).
- Triangulate against achieved BP: at ~27% stroke reduction per 10 mmHg SBP, −3.6 mmHg predicts ~10% at population level and −8.1 mmHg ~20% among hypertensives — coherent with the observed 16–25%, and evidence that the 40% design assumption was unattainable.
- Discuss substitution of public-system care (PHC use 24.3% control vs 3.1% intervention at endline) and the tobacco pamphlet as an active co-intervention in the control arm.

**Reproducibility**

 The IPD restriction is understandable and declared.

**Literature and corrections**

- CRHCP https://pubmed.ncbi.nlm.nih.gov/35500594/  village-doctor-led care, 23 mmHg net SBP difference, ~33% lower CVD death
- GBD 2021 stroke https://pubmed.ncbi.nlm.nih.gov/39304265/   for the per-10-mmHg benchmark.
- Table 1 CI impossible (52.3% [54.4, 53.1]); baseline stroke mortality sentence duplicated and miscited to Figure 3 instead of Figure 4; orphaned "(p = 0.003)…" fragment; Discussion 3.5/2.4 mmHg vs Results 3.6/2.3; salt p=0.005 in text vs "p = 0.00" in Table 3; "hazard ration"; "Randomiszation"; Figure 1 is a CONSORT not PRISMA diagram; supplementary numbering S10–S14 vs list ending at S13; reference 14 lacks an identifier.
- **Summary: **The trial shows a multicomponent CHW-supported program is deliverable at over 90% coverage with 85–90% adherence in a challenging Indian setting, with gains in treatment coverage, blood pressure, and awareness. The mortality outcome is inconclusive, as effect size assumptions exceeded BP differences, baseline event rates were lower than expected, and misclassification likely shrank the apparent mortality reduction from 25% to 16%.

Is the work clearly and accurately presented and does it cite the current literature?

Yes

If applicable, is the statistical analysis and its interpretation appropriate?

Yes

Are all the source data underlying the results available to ensure full reproducibility?

Yes

Is the study design appropriate and is the work technically sound?

Yes

Are the conclusions drawn adequately supported by the results?

Yes

Are sufficient details of methods and analysis provided to allow replication by others?

Yes

Reviewer Expertise:

NCD Prevention, Epidemiology, Trial design and analysis

I confirm that I have read this submission and believe that I have an appropriate level of expertise to confirm that it is of an acceptable scientific standard.

# Reviewer report: Reviewer response for version 1

**DOI:** 10.21956/wellcomeopenres.28619.r157579

**Published:** 2026-06-24

**Authors:** Jiale Zhang

**Affiliations:** 1 China Academy of Chinese Medical Sciences, Beijing, China

The trial was powered to detect a 40% reduction in stroke mortality over the final 2.5 years, but the observed reduction was 16% (HR 0.84, p=0.23). The post-hoc analysis showing a 25% reduction over the entire 3.5 years (HR 0.75, p=0.022) is intriguing but should be clearly labelled as exploratory, as it was not pre-specified. I recommend that the Abstract and Conclusions place greater emphasis on the negative primary outcome, while acknowledging the post-hoc finding as hypothesis-generating. The current phrasing ("there was a reduction... but this reduction was not significant") may inadvertently overstate the clinical certainty of benefit.

 The authors acknowledge limited access to brain imaging as a limitation. From a clinical standpoint, this is particularly relevant for the use of aspirin in secondary prevention without CT confirmation. While the authors cite large studies suggesting safety, the risk of giving aspirin to patients with undiagnosed intracerebral haemorrhage remains a real clinical concern in this setting. I would encourage the authors to elaborate on how they clinically excluded haemorrhagic stroke, and whether any adverse events related to aspirin use were systematically monitored and reported.

 The gradual rollout of the NP-NCD in control villages during the trial period is an important confounder. The authors note that screening and treatment access improved in the control arm, which may have attenuated the observed mortality difference. This is a strength of the trial design (it reflects real-world conditions), but it also means the intervention effect may be underestimated. I would suggest that the Discussion more explicitly quantify the potential dilution effect—for example, by estimating the proportion of control participants who received NP-NCD services—and comment on how this might inform the interpretation of the non-significant primary outcome.

 The intervention involved monthly home visits by CHWs, outreach physician visits every 2–4 months, and free medications delivered at the village level. This level of intensity may be difficult to sustain within routine public health systems, even if it is clinically effective. The authors argue that such intensity is justified in low-literacy settings, which is reasonable. However, I would appreciate a more pragmatic discussion of how this model could be scaled with existing human resources (e.g., one ASHA per village is already overburdened with maternal and child health duties). What is the minimum effective dose of the intervention that could be realistically integrated into the NP-NCD?

 Minor points:

 The trial registration (CTRI/2015/12/006424) is provided, but the authors should confirm that all pre-specified outcomes are reported in accordance with the protocol.

 The supplementary tables are comprehensive, but some key figures (e.g., medication compliance over time, adverse event rates) would be helpful in the main text to support the claim that the intervention was acceptable and safe.

 The delay in publication is acknowledged. I appreciate the transparency, and the reasons provided (COVID-19, institutional changes) are understandable. The results remain relevant given the ongoing scale-up of NP-NCD in India.

 Overall conclusion: This is a commendable effort in a challenging setting. The trial demonstrates that a community-based, task-shared intervention is feasible and acceptable, and it improves blood pressure control and awareness. The lack of a statistically significant reduction in the primary outcome should not be seen as a failure—it provides important lessons for future trial design, including the need for longer intervention periods, larger sample sizes, and accounting for concurrent policy changes. The findings will inform the ongoing refinement of non-communicable disease programmes in India and other LMICs.

Is the work clearly and accurately presented and does it cite the current literature?

Yes

If applicable, is the statistical analysis and its interpretation appropriate?

Partly

Are all the source data underlying the results available to ensure full reproducibility?

Yes

Is the study design appropriate and is the work technically sound?

Yes

Are the conclusions drawn adequately supported by the results?

Yes

Are sufficient details of methods and analysis provided to allow replication by others?

Yes

Reviewer Expertise:

cardiovascular disease

I confirm that I have read this submission and believe that I have an appropriate level of expertise to confirm that it is of an acceptable scientific standard, however I have significant reservations, as outlined above.

## References

[1] : The Stroke Burden in China and Its Long-Term Trends: Insights from the Global Burden of Disease (GBD) Study 1990-2021. *Nutrition, Metabolism and Cardiovascular Diseases* .2025;35(7) : 10.1016/j.numecd.2025.103848 10.1016/j.numecd.2025.103848 39948019

# Response

**Published:** 2026-06-29

**Authors:** Yogeshwar Kalkonde

**Affiliations:** Sangwari-People's Association for Equity and Health, India

The trial was powered to detect a 40% reduction in stroke mortality over the final 2.5 years, but the observed reduction was 16% (HR 0.84, p=0.23). The post-hoc analysis showing a 25% reduction over the entire 3.5 years (HR 0.75, p=0.022) is intriguing but should be clearly labelled as exploratory, as it was not pre-specified. I recommend that the Abstract and Conclusions place greater emphasis on the negative primary outcome, while acknowledging the post-hoc finding as hypothesis-generating. The current phrasing ("there was a reduction... but this reduction was not significant") may inadvertently overstate the clinical certainty of benefit. ***Re: We have now reworded the lines in the abstract as – “After the intervention, the stroke mortality rate per 100,000 person-years in the last 2.5 years of the intervention, which was the primary outcome of the trial, was comparable in the intervention (416.7) and the control arm (484.9) (adjusted hazard ration [HR] 0.84, 95 % confidence interval [0.62,1.12]; p=0.23)”.  The abstract and Conclusions already highlight negative primary outcome. We have not included the description of post-hoc analysis in the abstract. We have also edited the results section to highlight negative primary outcome.***

 The authors acknowledge limited access to brain imaging as a limitation. From a clinical standpoint, this is particularly relevant for the use of aspirin in secondary prevention without CT confirmation. While the authors cite large studies suggesting safety, the risk of giving aspirin to patients with undiagnosed intracerebral haemorrhage remains a real clinical concern in this setting. I would encourage the authors to elaborate on how they clinically excluded haemorrhagic stroke, and whether any adverse events related to aspirin use were systematically monitored and reported. ***Re: Stroke patients who had loss of consciousness or seizures at the onset of symptoms were presumed to have had a haemorrhagic stroke and were not given aspirin. We have reported this information in the trial protocol but have added in the procedure section of the manuscript now. As a precautionary measure, we did monitor adverse events related to aspirin use among all users. These were systematically recorded and monitored by the external Data and Safety Monitoring Committee. No excessive adverse events were observed. We have now added this information to the limitations section under discussions. Supplementary table S12 also provides information on serious adverse events among patients with stroke.***

 The gradual rollout of the NP-NCD in control villages during the trial period is an important confounder. The authors note that screening and treatment access improved in the control arm, which may have attenuated the observed mortality difference. This is a strength of the trial design (it reflects real-world conditions), but it also means the intervention effect may be underestimated. I would suggest that the Discussion more explicitly quantify the potential dilution effect—for example, by estimating the proportion of control participants who received NP-NCD services—and comment on how this might inform the interpretation of the non-significant primary outcome. ***Re: We appreciate the reviewer's suggestion. We agree that exposure to services under the National Programme for Prevention and Control of Non-Communicable Diseases (NP-NCD) could potentially attenuate the observed intervention effect. However, we were unable to quantify this dilution because exposure to NP-NCD services cannot be meaningfully classified as a binary variable. NP-NCD comprises a basket of services across the continuum of care like screening, referral, diagnosis and treatment. The care seeking in the real world was dynamic rather than linear. For example, a participant in the control arm may have been screened through NP-NCD but subsequently diagnosed and treated by a private provider; another may have been diagnosed by a private provider and later received treatment through NP-NCD; while others may have been referred by NP-NCD but never completed the referral pathway. Also, the surveys in the trial were not designed to capture individual-level exposure to routine NP-NCD services, making retrospective estimation unreliable. Hence, while we agree with the reviewer that efforts should be made to quantify the effect of NP-NCD, it is difficult to reliably estimate the proportion of control participants who 'received NP-NCD services' using the available data. We have now clarified this part in the limitations part in the Discussion section.***

 The intervention involved monthly home visits by CHWs, outreach physician visits every 2–4 months, and free medications delivered at the village level. This level of intensity may be difficult to sustain within routine public health systems, even if it is clinically effective. The authors argue that such intensity is justified in low-literacy settings, which is reasonable. However, I would appreciate a more pragmatic discussion of how this model could be scaled with existing human resources (e.g., one ASHA per village is already overburdened with maternal and child health duties). What is the minimum effective dose of the intervention that could be realistically integrated into the NP-NCD? ***Re:*** ***We thank the reviewer for these comments.*** ***The intervention evaluated in this study was deliberately intensive, incorporating monthly community health worker visits, periodic physician outreach, and village-level medication delivery to address well-recognised barriers to chronic disease care in rural, low-literacy settings. Also, our study was designed to evaluate the effectiveness of the intervention package rather than to identify the contribution of individual components. Determination of minimum effective dose that could be integrated into NP-NCD would need further implementation research which can explore making medications available in villages, risk-stratified follow-ups (e.g. those with blood pressure >160/100 or those who have both hypertension and diabetes, those with poor medication compliance), reduced visit frequency by the CHWs after initial disease control, greater use of facility-based NP-NCD services for stable patients. Such optimisation studies would help identify delivery models that balance effectiveness with feasibility within existing health system resources. We have now added a few lines in the Discussion section to elaborate more on this point.***   Minor points:

 The trial registration (CTRI/2015/12/006424) is provided, but the authors should confirm that all pre-specified outcomes are reported in accordance with the protocol. ***Re: Thank you. We have now confirmed this in the last paragraph of the Methods section.***

 The supplementary tables are comprehensive, but some key figures (e.g., medication compliance over time, adverse event rates) would be helpful in the main text to support the claim that the intervention was acceptable and safe. ***Re: Thank you. We have now moved figures on medication compliance and serious adverse events to the main text.***

 The delay in pu blication is acknowledged. I appreciate the transparency, and the reasons provided (COVID-19, institutional changes) are understandable. The results remain relevant given the ongoing scale-up of NP-NCD in India. ***Re: We thank the reviewer for these positive comments.***

 Overall conclusion: This is a commendable effort in a challenging setting. The trial demonstrates that a community-based, task-shared intervention is feasible and acceptable, and it improves blood pressure control and awareness. The lack of a statistically significant reduction in the primary outcome should not be seen as a failure—it provides important lessons for future trial design, including the need for longer intervention periods, larger sample sizes, and accounting for concurrent policy changes. The findings will inform the ongoing refinement of non-communicable disease programmes in India and other LMICs. ***Re: We thank the reviewer for the overall positive comments on the study.***

# Reviewer report: Reviewer response for version 1

**DOI:** 10.21956/wellcomeopenres.28619.r157585

**Published:** 2026-06-23

**Authors:** Jeemon Panniyammakal

**Affiliations:** 1 Sree Chitra Tirunal Institute for Medical Sciences and Technology, Thiruvananthapuram, Kerala, India

Conducting a large cluster randomised controlled trial in resource-poor settings to evaluate a community health worker-led intervention for stroke prevention is a remarkable achievement. Such trials are methodologically challenging and require extensive coordination across communities and health systems. The study provides robust evidence for scalable, community-based strategies that have the potential to substantially reduce stroke mortality in the long-term and improve population health in underserved settings.

 I have the following comments: 

 1. Provide reasons for excluding 9 clusters from randomisation in the PRISMA flow chart. Explain it in the manuscript text. 

 2. Primary outcome data: Please clearly mention whether you have primary outcome data from the migrated individuals. If yes, modify the numbers accordingly. 

 3. The statistician involved in the randomisation and conducted the analysis: Is it the same individual? Please clearly explain the independent nature of the randomisation procedure. 

 4. When was the randomisation done? Is it after the completion of all baseline data collection from all clusters? Please explain. 

 5. Who collected information on mortality and administered the VA tool? Explain the VA technique, analysis and final validation procedures. 

 6. Describe the intervention using TIDieR checklist and provide it in the online supplement. 

 7. What was the cluster size assumed in the sample size calculation? Did you include a term for variation in cluster size?

Is the work clearly and accurately presented and does it cite the current literature?

Yes

If applicable, is the statistical analysis and its interpretation appropriate?

I cannot comment. A qualified statistician is required.

Are all the source data underlying the results available to ensure full reproducibility?

No

Is the study design appropriate and is the work technically sound?

Yes

Are the conclusions drawn adequately supported by the results?

Yes

Are sufficient details of methods and analysis provided to allow replication by others?

Yes

Reviewer Expertise:

Cardiovascular disease prevention and control

I confirm that I have read this submission and believe that I have an appropriate level of expertise to confirm that it is of an acceptable scientific standard.

# Response

**Published:** 2026-06-29

**Authors:** Yogeshwar Kalkonde

**Affiliations:** Sangwari-People's Association for Equity and Health, India

Conducting a large cluster randomised controlled trial in resource-poor settings to evaluate a community health worker-led intervention for stroke prevention is a remarkable achievement. Such trials are methodologically challenging and require extensive coordination across communities and health systems. The study provides robust evidence for scalable, community-based strategies that have the potential to substantially reduce stroke mortality in the long-term and improve population health in underserved settings. ***Re: We thank the reviewer for these positive comments.***

 I have the following comments: 

 1. Provide reasons for excluding 9 clusters from randomisation in the PRISMA flow chart. Explain it in the manuscript text. ***Re: There were 73 eligible clusters available. The study protocol required only 64 clusters. A computer randomly selected 64 of the 73. The remaining 9 were simply not selected for inclusion. We have now edited the PRISMA flow chart and specified that they were not selected for inclusion.***

 2. Primary outcome data: Please clearly mention whether you have primary outcome data from the migrated individuals. If yes, modify the numbers accordingly. ***Re: Thank you for pointing this out. We do not have primary outcome data on migrated individuals. We have now specified this clearly in the Results section of the manuscript.***

 3. The statistician involved in the randomisation and conducted the analysis: Is it the same individual? Please clearly explain the independent nature of the randomisation procedure. ***Re: The statistician involved in randomisation and analyses were different. We have now specified this in the Randomization and blinding section under Methods.***

 4. When was the randomisation done? Is it after the completion of all baseline data collection from all clusters? Please explain. **Re: Randomization was performed before baseline data collection. Baseline data were collected in randomized villages. We have now specified this in *the Randomization and blinding section under Methods.***  

 5. Who collected information on mortality and administered the VA tool? Explain the VA technique, analysis and final validation procedures. **Re: A team of trained field supervisors who were separate from the implementation team administered the VA tool. We have now described the VA technique, analysis and validation procedure in the Outcomes section under the Methods and added one reference for clarity.**

 6. Describe the intervention using TIDieR checklist and provide it in the online supplement. ***Re: We have now described the intervention using the TIDieR checklist and provided it as an online supplement.***

 7. What was the cluster size assumed in the sample size calculation? Did you include a term for variation in cluster size? ***Re: The cluster size assumed in the sample size calculation was 1200 with 16% of the cluster population above the age of 50 based on prior data from the demographic surveillance system. We used between-cluster correlation coefficient of variation to account for variation in cluster size as described in the Methods section.***
